# Condition-Dependent Noise Correlations without Condition-Dependent Spike Counts

**DOI:** 10.64898/2026.05.08.723078

**Published:** 2026-05-20

**Authors:** Doyeon Kim, Matthew F. Panichello, Tirin Moore

**Affiliations:** 1 Department of Bioengineering, Stanford University; 2 Department of Neurobiology and Howard Hughes Medical Institute, Stanford University

## Abstract

The ability of the brain to encode information and control behavior depends on the coordinated activity of large and distributed neuronal populations. Correlations in neuronal spiking activity across trials of the same condition, or noise correlations (NCs), have been interpreted as a reflection of shared synaptic connectivity and as a contributing factor to the information capacity of neuronal populations. The impact of NCs on coding is most often considered in populations of neurons exhibiting robust condition-dependent information in their spike counts (SCs). However, theoretical work suggests that NCs could provide a source of condition-dependent information separate from SCs. We examined the activity of large neuronal populations in prefrontal cortex of macaques while they performed a spatial delayed response task composed of visual, memory, and motor epochs. We found that pairs of neurons that displayed visual, memory, and motor selectivity in their SCs often exhibited selectivity in their NCs, independent of spike count. However, we also found that pairs of neurons without SC selectivity during the different task epochs nonetheless exhibited condition-dependent NCs. Moreover, we found that the magnitude of condition-dependent NCs were largely comparable across neuronal pairs with or without SC selectivity. These results demonstrate that correlated variability in spiking activity can be condition-dependent even in the absence of condition-dependent SCs.

## Introduction

Representations of information by the brain have largely been characterized through the lens of neuronal firing rates. However, relying solely on trial-averaged firing rates offers an incomplete picture of population coding, as this approach assumes that neurons act as independent encoders, thereby ignoring the complex, moment-to-moment fluctuations that are ubiquitous in neural circuits (Panzeri et al., 2015). Information can be encoded not just in the mean rate but in the patterns of joint activity, which are captured by second-order statistics (Scaglione et al., 2011; Vaadia et al., 1995). For example, when an identical stimulus is presented repeatedly, trial-to-trial fluctuations in the response of one neuron are often correlated with those of others. These correlations represent shared variability that cannot be explained by the mean response to the stimulus (Ponce-Alvarez et al., 2013). Coordinated variability in the firing rates of populations of neurons has been linked to neural computations associated with myriad brain functions (Zavitz & Price, 2019), such as sensory coding (Bondy et al., 2018; Kohn & Smith, 2005; Singer, 1999), attention (Downer et al., 2017), and decision-making (Nassar et al., 2021). These correlations in neuronal spike counts (SCs), or noise correlations (NCs), have been interpreted as a reflection of shared synaptic connectivity and as a contributing factor to the information capacity of neuronal populations.

Previous studies have established that NCs among pairs of neocortical neurons often depend on the similarities in the selective properties of the constituent neurons (Hofer et al., 2011), consistent with the supposition that NCs reflect shared connectivity. In addition, other studies have revealed that for given neuronal pairs, NCs also vary systematically across conditions, e.g. across different sensory stimuli (Ponce-Alvarez et al., 2013), an effect that could increase the information encoded in population activity that is complementary to neuronal firing rates (Josić et al., 2009; Pola et al., 2003; Panzeri et al., 1999; Shamir et al., 2004). However, to the extent that condition-dependent NCs contribute to population coding, that contribution has largely been considered in populations of neurons already conveying robust condition-dependent information in their firing rates, e.g. stimulus selective neurons (Franke et al., 2016; Zylberberg et al., 2016). Although condition-dependent NCs among populations of neurons without SC selectivity could also contribute to population coding, little or no empirical evidence of that dependence exists. The role of weakly- or non-SC selective neurons in neural computations and population coding, while less clear than that of SC selective neurons, has nonetheless become apparent in some neural systems. For example, in the mammalian visual system, the contribution of inhibitory interneurons to stimulus coding, e.g. orientation tuning, is well-established (Wood et al., 2018), yet these neurons are also known to be weakly tuned compared to excitatory neurons (Hofer et al., 2011; Kerlin et al., 2010; Runyan et al., 2013; Scholl et al., 2015), at least in part due to more extensive local connectivity (Hofer et al., 2011; Hu et al., 2012; Scholl et al., 2015). Similarly, neurons in prefrontal cortex exhibiting little or no SC selectivity during short-term memory tasks appear to contribute nonetheless to task performance (Aliramezani et al., 2025; Kim et al., 2016). Thus, it should be informative to assess the condition-dependence of NCs among neurons with non-selective SCs to various stimulus and task variables.

Building on these findings, we sought to determine the extent to which NCs provide a source of task-relevant information that is decoupled from individual neuronal tuning within the macaque lateral prefrontal cortex (LPFC). By recording from large neuronal populations during a spatial delayed response task, we investigated the degree to which NCs are modulated across distinct visual, mnemonic, and motor epochs. Specifically, we aimed to characterize the selectivity of NCs not only in neurons with robust SC selectivity but also in populations in which such selectivity is weak or absent. Below, we briefly summarize our results to date.

## Results

**Figure 1.**
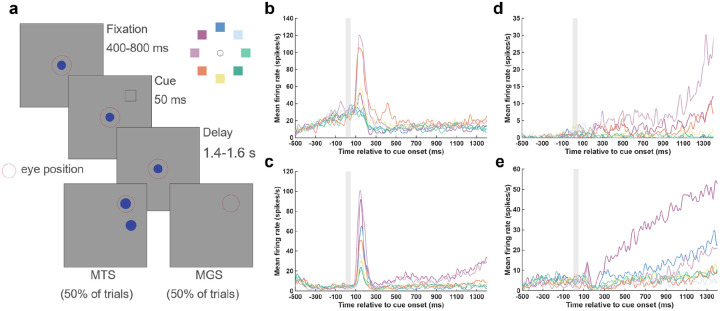
Task paradigm and functional diversity of prefrontal neurons. **a,** Schematic of the delayed Match-to-Sample (MTS) and Memory-Guided Saccade (MGS) tasks. On each trial, a cue was presented at one of eight possible locations (inset). Following a memory delay epoch, the animal received juice as the reward for making an eye movement to the previously cued location (red dashed circles indicate eye position). **b-e,** Trial-averaged peristimulus time histograms (PSTHs) for four example prefrontal neurons exhibiting diverse SC selectivity across task epochs. Grey vertical bars indicate the cue presentation epoch (0–50 ms). Example neurons with condition-dependent activity during **b**, the Visual epoch, **c**, both the Visual and Delay epochs, **d-e,** the Delay epoch. Cue locations are color-coded as shown in the inset of panel a.

We analyzed the spiking activity of large populations of neurons from LPFC using Neuropixels probes of three macaque monkeys (Monkeys A, H, and J)(Panichello et al., 2024). During recordings, monkeys performed one of two versions of an oculomotor spatial delayed response task. In either version of the task, on each trial, one of eight possible peripheral locations was cued (Figure 1a), followed by a delay epoch during which the monkey needed to maintain the location in working memory. The trial concluded with a reward if the monkey made a saccadic eye movement response either to the remembered location or to one of two appearing targets that matched the cued location. In each recording session, we simultaneously measured the spiking activity of hundreds of neurons (mean ± SEM, 329 ± 46; total n = 8,225 units across 25 sessions) across LPFC (area 8/9/46, see Methods).

We first assessed the SC selectivity of individual neurons in each of the task epochs by comparing their responses across cue locations during the presentation of the visual cue (“Visual”; 0–300 ms post-cue onset), memory delay (“Delay”; 400–1,200 ms post-cue), and the saccadic response (“Motor”; −125–0 ms relative to movement onset) (see Methods). As in previous studies (Bruce & Goldberg, 1985; Funahashi et al., 1989), neurons showed a variety of SC selectivity in the Visual, Delay and Motor epochs (Figure 1, b–e). Across the recording sessions, a large proportion of neurons showed SC selectivity for each of the epochs (Visual, 62.98 ± 5.79 %; Delay, 55.65 ± 5.21 %; Motor, 66.89 ± 5.53 %) (mean ± SEM). For example, the neuron shown in Figure 1b exhibited SC selectivity during the Visual epoch but was non-selective during the Delay epoch. Other neurons exhibited selective responses during a different epoch, such as the Delay epoch (Figure 1 d–e). These observations highlight the well-known observation that neurons do not necessarily display selectivity across all task epochs but can display different combinations of Visual, Delay, and Motor selectivity in their SCs.

**Figure 2.**
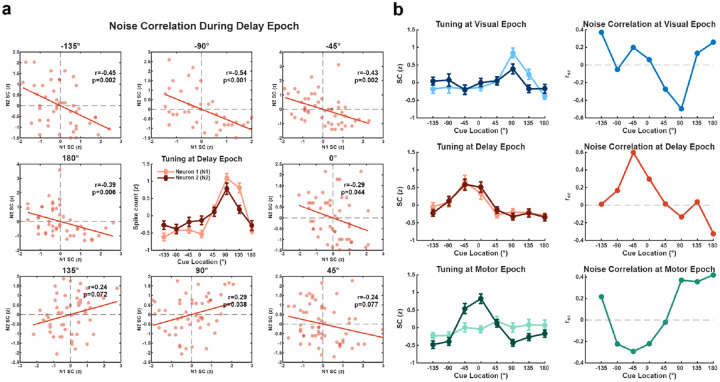
NCs and SC tuning curves for neuronal pairs. **a,** Example of condition-dependent modulation of NCs in a neuron pair during the Delay epoch. The central subplot displays the SC tuning curves of two neurons across cue locations. Error bars represent standard error of the mean (SEM). Surrounding subplots show the trial-to-trial scatter of z-normalized SCs for the two neurons at each corresponding cue location. Solid lines indicate linear regression fits. Pearson correlation coefficients (r) and p-values are indicated in the top right for each cue location. For this example pair, regression analysis revealed a significant modulation of NCs by cue location (p < 0.001). **b,** Examples of selectively tuned neuron pairs recorded during the Visual (top), Delay (middle), and Motor (bottom) epochs. Left column: tuning curves with SCs (z-scored) of the two neurons across eight cue locations. Right column: Pairwise NC (r_sc_) values for each corresponding neuron pair from the left column, across the same cue locations. For all three examples, a significant modulation of NCs by cue location was observed (all p < 0.001).

We next assessed whether NCs were condition-dependent by examining pairs of neurons with SC tuning to cue location (see Methods). Figure 2a illustrates a representative pair of SC selective neurons during the Delay epoch. The central subplot shows that both neurons exhibited distinct SC tuning across the eight different cue locations. In other words, their SCs were condition-dependent. The surrounding scatter plots illustrate that the NCs between these neurons were not constant but varied systematically with the cue location, suggesting that NCs were also condition-dependent.

To identify pairs in which NCs carried information about the cue, we z-normalized the SCs for each neuron in a pair within each cue location to remove the effect of cue location on mean SC, then modeled their relationship using linear regression as:

$$N_{2}\sim \beta_{0}+\beta_{1}N_{1}+\beta_{2}(N_{1}\times C)$$

where N_2_ is the normalized SC of one neuron, N_1_ is the normalized SC of the other, and C is a categorical coding of cue location. A significant interaction term (p < 0.05) thus indicates that the NC between neurons is modulated by cue location. For example, for the neuronal pair in Figure 2a, the regression analysis revealed a significant modulation of NCs by cue location during the Delay epoch (p < 0.001).

Next, we extended this analysis to different task epochs. As shown in the example pairs in Figure 2b, we observed condition-dependent NCs across the Visual, Delay, and Motor epochs. In these examples, SC tuning curves showed varying degrees of selectivity depending on the task epoch. In addition, NCs also depended on the cue location, and regression analyses revealed a significant modulation of NCs by cue location in each case (Figure 2b; Visual and Motor epochs, p < 0.001 Delay epoch, p < 0.05).

**Figure 3.**
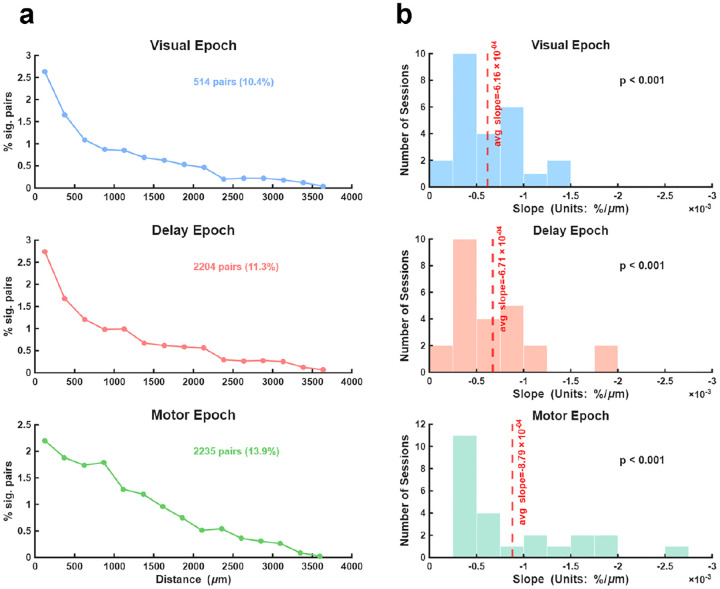
Spatial organization of condition-dependent NCs. **a,** Percentage of SC selective neuron pairs exhibiting significant condition-dependent modulation of NCs (interaction term, p < 0.05) as a function of anatomical distance (μm) between pairs for an example session. **b,** Histograms display the distribution of slopes (%/μm) derived from the linear relationship between the percentage of significant pairs and distance between neuronal pairs for all sessions (N=25). Red dashed lines indicate the average slope across sessions.

Having observed condition-dependent NCs in individual neuronal pairs, we next assessed the extent to which that effect depended on the distance between neuronal pairs. We hypothesized that since NCs typically scale with the distance between neurons (Shapcott et al., 2016; Shilling-Scrivo et al., 2022; Smith & Kohn, 2008) so too should the prevalence of condition-dependent NCs. Indeed, we observed a clear distance-dependent decline in the example session (Figure 3a): linear regression analysis revealed that neurons in closer proximity were significantly more likely to share condition-dependent NCs across all three epochs (Visual, Delay, and Motor all p < 0.001). To assess the effect of neuronal distance across the entire population, we quantified the slope of the percentage-distance relationship (%/μm) for each session. Figure 3b displays the distribution of these slopes for each epoch. A left-tailed Wilcoxon signed-rank test confirmed that the distribution of slopes was significantly shifted toward negative values for the Visual, Delay, and Motor epochs (all p < 0.001), with the percentage of significant pairs (Visual, 11.33 ± 0.82%; Delay, 11.53 ± 1.01 %; Motor, 14.71 ± 1.37 %) (mean ± SEM) exceeding that expected by chance (Wilcoxon signed-rank test, all p < 0.001). This result indicates that condition-dependent NCs were confined to nearby neurons.

**Figure 4.**
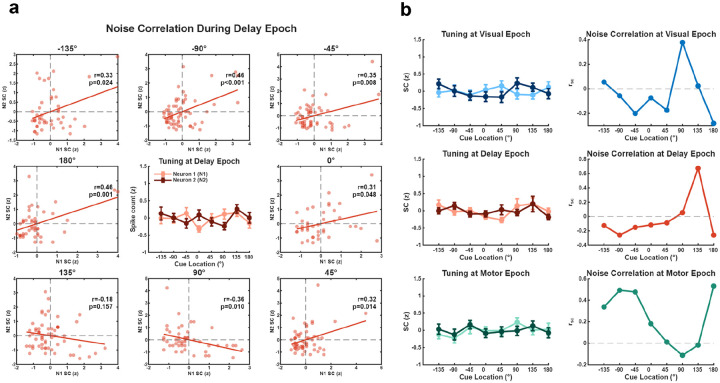
NCs and SC tuning curves for non-selective neuron pairs. **a,** Example of condition-dependent modulation of NCs in a non-selective neuron pair during the Delay epoch. The central subplot displays the SC tuning curves of two neurons across eight cue locations (error bars, SEM). Surrounding subplots show trial-to-trial scatterplots of z-normalized SCs for each cue location. Solid lines indicate linear regression fits, with Pearson correlation coefficients (r) and p-values provided for each panel. For this pair, regression analysis revealed a significant modulation of NCs by cue location (p < 0.001). **b,** Examples of non-selective neuron pairs across task epochs (top: Visual, middle: Delay, bottom: Motor). Left column: tuning curves with SCs (z-scored) across eight cue locations, showing relatively flat tuning for both neurons. Right column: Pairwise noise correlation (r_sc_) values for the corresponding pairs from the left column. For all examples, regression analysis revealed a significant modulation of NCs by cue location (Visual (p = 0.0428); Delay (p < 0.001); Motor epochs (p < 0.001)).

We next asked if condition-dependent NCs were confined to SC selective neurons by performing the above analyses on non-selective neuron pairs, i.e. those lacking significant SC tuning. Figure 4a shows two representative neurons that were non-selective during the Delay epoch. However, despite the absence of delay tuning, NCs varied across cue location conditions. The interaction term in the regression model confirmed this condition-dependent modulation in NCs for this pair (p < 0.001). Notably, we observed condition-dependent NCs in non-selective neuronal pairs in all three task epochs (Figure 4b). For the example cases in Figure 4b, regression analyses revealed a significant modulation of NCs by cue location (Visual, p = 0.0428; Delay, p < 0.001 Motor, p < 0.001).

**Figure 5.**
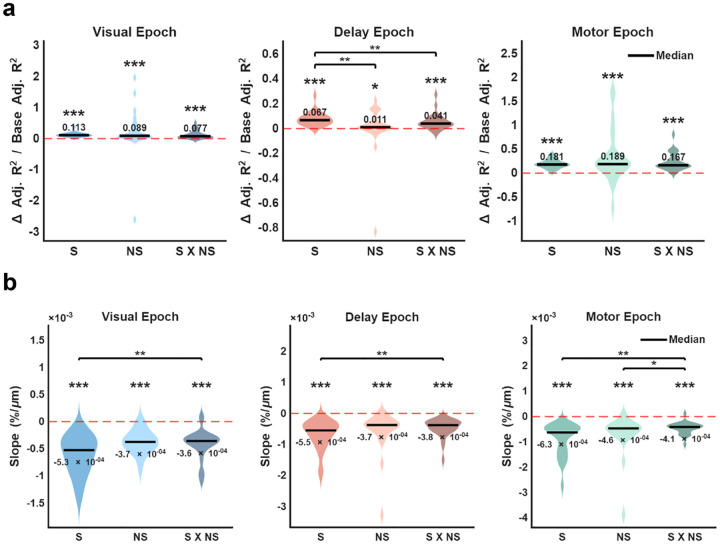
Population-level analyses of condition-dependent NCs in selective, non-selective and mixed neuronal pairs. **a,** Violin plots show the proportional increase in variance explained by condition-dependent NCs, calculated as the normalized change in adjusted R^2^ (see Methods). Each data point within the violin plots represents an individual session, excluding one outlier session with a magnitude > 5 in the Visual epoch. **b,** Violin plots show the slopes of the percentage of significant pairs as a function of distance (%/μm) across all sessions. **a-b,** Data are categorized into three groups: Selective pairs (S), Non-selective pairs (NS), and Mixed pairs (S × NS). Significant differences between groups are indicated by horizontal bars. Horizontal black lines for each group indicate the median. Asterisks denote significance (*, p < 0.05; **, p < 0.01; ***, p < 0.001).

To quantify the magnitude of NC effects in the full population of neuronal pairs in which both neurons were selective (S), both neurons were non-selective (NS), or pairs in which one neuron was selective and one was non-selective (Mixed) based on their SCs, we measured the proportional increase in variance explained by condition-dependent NCs (Figure 5a). We did this by computing the proportional change in adjusted R^2^ for the full regression model compared to a base model without the condition-dependent interaction term (see Methods). All S, NS, and Mixed pairs showed significantly positive values across all three epochs, (Wilcoxon signed-rank test, right-tailed, p < 0.05), confirming that NCs were condition-dependent at the population level. In the Visual and Motor epochs, the proportional increase in variance explained did not differ significantly across the three groups (all pairwise comparisons, p > 0.05). In contrast, during the Delay epoch, the proportional change in adjusted R^2^ was greater for S pairs than NS pairs (p = 0.0080) and Mixed pairs (p = 0.0038).

To examine the effect of distance on the NC effects across the entire population of neuronal pairs, we measured the decline in condition-dependent NCs for pairs in each of the task epochs (Figure 5b). Slopes were significantly negative across all three groups and all task epochs (Wilcoxon signed-rank test, left-tailed, p < 0.001). In the Visual and Delay epochs, the slopes of S pairs were significantly different from those of Mixed pairs (Wilcoxon signed-rank test, Visual: p = 0.0035; Delay: p = 0.0013), while no significant differences were found between S and NS pairs or between NS and Mixed pairs (p > 0.05). In the Motor epoch, Mixed pairs showed significantly different slopes compared to both S (p = 0.0043) and NS pairs (p = 0.0124), although S and NS pairs were similar (p = 0.3317). Between different epochs, S pairs exhibited significant differences in their slopes when comparing the Motor epoch to other epochs (Wilcoxon signed-rank test, Visual vs Motor epoch: p = 0.0039; Delay vs Motor epoch: p = 0.0036), while the difference between the Visual and Delay epoch was not significant (p = 0.4758). Non-selective pairs showed a significant difference only between the Delay and Motor epoch (p = 0.0081), while Mixed pairs did not show significant differences across all epochs (p > 0.05). These results indicate small, but reliable variation in the spatial extent of condition-dependent NCs in the different types of neuronal pairs.

## Discussion

In this study, we investigated the prevalence and spatial dependence of condition-dependent NCs in the LPFC during a spatial delayed response task composed of visual, memory, and motor epochs. By simultaneously measuring the spiking activity of hundreds of neurons, we found that a significant population of neuronal pairs exhibited robust, condition-dependent modulations of NCs across Visual, Delay, and Motor epochs. Critically, while previous studies have largely focused on condition-dependent NCs between neurons with SC selectivity, we observed a condition-dependent modulation of NCs of comparable magnitude between nonselective neurons. These condition-dependent NCs showed a distinct spatial dependence as well, with pairs in closer proximity more likely to exhibit such modulations. These results demonstrate that correlated variability in spiking activity can be condition-dependent even in the absence of condition-dependent SCs. Our findings suggest that NCs are not merely a byproduct of shared synaptic inputs among tuned neurons, but a robust feature of the population that can serve as a distinct channel for task-relevant information.

## Methods

### Animal Subjects

Data were collected from three male rhesus monkeys (Macaca mulatta), designated as A (11 years, 11 kg), H (12 years, 14 kg), and J (8 years, 12 kg), as previously reported (Panichello et al., 2024). All experimental protocols and surgical procedures were performed in accordance with the National Institutes of Health guidelines and received approval from the Stanford University Institutional Animal Care and Use Committee (IACUC).

### Experimental Setup and Behavioral Task

Visual stimuli were displayed on a VIEWPixx3D monitor (60 cm viewing distance) by MATLAB (R2022a) and Psychtoolbox. Eye movements were recorded at 1 kHz using an Eyelink 1000 system. Trials began with the monkeys fixating on a central spot against a grey background for 600–800 ms. Subsequently, a square cue (green, black, or white for monkey A, H, and J, respectively) measuring 1 degree of visual angle (DVA) on a side appeared for 50 ms at one of eight possible locations (5–7 ° eccentricity, 45° spacing). After a variable memory delay (1,400–1,600 ms), the fixation spot was extinguished, cuing the response phase, which took one of two forms. On Match-to-Sample (MTS) trials, two targets (blue circles, 1 DVA radius) were presented: one at the cued location and another at a distractor location (one out of the remaining seven locations). On Memory-Guided Saccade (MGS) trials, no targets were shown. Monkeys A and H were trained on and performed randomly interleaved MTS and MGS trials, whereas Monkey J was trained on and completed only MGS trials. To receive a fluid reward, subjects had to saccade to within 5 DVA of the cued location and maintain gaze for 200 ms. Strict fixation (within 2 DVA of the fixation mark for monkeys H and J, within 3 DVA for monkey A) was required throughout the trial up until the response. Intertrial intervals (ITI) were 300–1,000 ms after correct trials, while fixation breaks and incorrect responses resulted in a 2,000 ms ITI without reward.

### Surgical Procedures and Electrophysiology

Subjects were implanted with titanium headposts for head stabilization and recording chambers for cortical access. Recordings were obtained from area 8 and the principal sulcus (9/46). Specifically, recordings were obtained from area 8 in monkey A, areas 8 and 9/46 in monkey H, and area 9/46 in monkey J. Neural activity was recorded using primate Neuropixels probes (384 active channels, 3.84 mm span). Probes were lowered through the dura via a 21-gauge cannula using custom 3 D-printed grids and motorized drives (NAN Instruments). To ensure stable recordings and mitigate probe drift, we allowed at least 30 minutes for the neurons to settle prior to data collection. Neural activity was monitored and saved using SpikeGLX (https://billkarsh.github.io/SpikeGLX/).

### Data Preprocessing and Analysis

Spike sorting was performed offline using Kilosort3, and both putative single- and multi-unit clusters were included in the analysis. Spike times were aligned to cue onset for each trial and binned with a width and timestep of 1 ms. Neurons that fired fewer than 1,000 spikes in the experimental sessions (each ~3 hours) were excluded from analysis.

### Statistical Standards

Except where noted otherwise, all statistical tests were two-sided. Task locations were randomized within each session to ensure balanced exposure for each subject within each session. Neurons were recorded without selection bias, with electrode placement optimizing for signal-to-noise ratio.

### Neuronal Classification via Population Decoding

We used multi-class linear decoders (support vector machines, as implemented by the fitcecoc function in MATLAB) to assess the amount of information about the cue location in neuronal firing rates. This analysis was performed separately for three task epochs: Visual (0–300 ms post-cue onset), Memory (400–1,200 ms post-cue), and Motor (−125–0 ms pre-saccade).For each session, spike counts were summed across each epoch on each trial and z-scored across all trials per neuron. Then, for each session and epoch, a decoder was trained to predict the cue location using a one-vs-one approach based on the vector of spike counts across the population. Overall classification performance was assessed using 10-fold cross-validation.

### Neuron Importance and Recursive Feature Ablation

To identify the contribution of individual neurons to the population representation, we analyzed the decoder weights. For each fold, we extracted the absolute weights (|W|) from all learners and averaged them across learners and folds to obtain an importance score for each neuron.

We then performed a neuron dropping analysis (recursive feature ablation). Units were ranked by their importance scores and sequentially removed from the population. After each removal, the 10- fold cross-validated decoding accuracy was recalculated using the remaining subset of neurons. This iterative process continued until all neurons were removed, allowing us to trace the decay of decoding performance as a function of unit loss.

To define a threshold for selectivity, we first calculated the 95% binomial confidence interval (CI) for the decoding accuracy at each removal step.

Next, we identified the point in the dropping analysis when decoding performance first dropped to chance level (i.e., when the CI included chance performance: 12.5% for 8 possible cue locations). All neurons removed prior to this point were classified as Selective, as their presence was necessary to maintain decoding accuracy above chance. Conversely, neurons remaining after this threshold were classified as Non-Selective, as their removal did not further degrade performance below the chance-level baseline. Some sessions never reached chance level and were therefore, not included in subsequent analysis. Finally, units were sorted into functional subclasses (e.g., Visual-selective, Delay-selective, or Motor selective) based on the specific epochs during which they met the selectivity criteria.

### Calculation of Pairwise Noise Correlations

To quantify noise correlations (r_sc_), spike counts for each unit and epoch were converted into z-scores by subtracting the mean and dividing by the standard deviation of spike counts across all correct trials for each cue location:

$$Z_{i,c}=\frac{x_{i,c}-\mu_{c}}{\sigma_{c}}$$

where *x*_*i,c*_ is the spike count on trial i for cue c, and μ_*c*_ and σ_*c*_ are the mean and standard deviation of spike counts for that cue, respectively. The noise correlation for a neuron pair was then defined as the Pearson correlation coefficient (r) between their z-scored spike counts across all trials for a specific cue location and epoch. Units that never fired during the task epoch of interest were excluded from analysis.

To investigate the spatial structure of noise correlations, we computed the center of mass of the spike waveform template for each unit using Neuropixels Utils (https://djoshea.github.io/neuropixel-utils/) and calculated the Euclidean distance between neurons in each pair.

### Pairwise Linear Regression Analysis

To evaluate the cue-dependent modulation of noise correlations, we employed linear regression, using MATLAB’s fitlm function. For each simultaneously recorded neuron pair, we modeled the z-scored spike count of one neuron (N_2_) as a function of the spike count of the other (N_1_) and its interaction with the cue location (C). We compared two nested models:

#### Base Model:


$$N_{2}\sim \beta_{0}+\beta_{1}N_{1}$$

#### Full Model:


$$N_{2}\sim \beta_{0}+\beta_{1}N_{1}+\beta_{2}(N_{1}\times C)$$

The Full Model specifically tested whether the slope of the relationship between N_1_ and N_2_ varied across the eight cue locations. Statistical significance of the interaction term was determined using an ANOVA (F-test) comparing the two models. All firing rates were z-score normalized within each session to account for differences in baseline excitability.

The improvement in model fit provided by the interaction term was quantified using the adjusted R-squared (Adj. R^2)^ to account for the increased number of predictors in the full model. We calculated the Normalized Information Metric as:

$$\text{Information}\;\text{Gain}=\frac{\text{Adj}.{R^{2}}_{\text{full}}-\text{Adj}.{R^{2}}_{\text{base}}}{\text{Adj}.{R^{2}}_{\text{base}}}$$

This metric represents the proportional increase in explained variance attributable to the cue-dependent modulation of noise correlations beyond a simple stimulus-independent correlation.
